# Evolving generalists in switching rugged landscapes

**DOI:** 10.1371/journal.pcbi.1007320

**Published:** 2019-10-01

**Authors:** Shenshen Wang, Lei Dai

**Affiliations:** 1 Department of Physics and Astronomy, University of California, Los Angeles, Los Angeles, California, United States of America; 2 Shenzhen Institute of Synthetic Biology, Shenzhen Institutes of Advanced Technology, Chinese Academy of Sciences, Shenzhen, China; University of Texas Southwestern Medical Center, UNITED STATES

## Abstract

Evolving systems, be it an antibody repertoire in the face of mutating pathogens or a microbial population exposed to varied antibiotics, constantly search for adaptive solutions in time-varying fitness landscapes. Generalists refer to genotypes that remain fit across diverse selective pressures; while multi-drug resistant microbes are undesired yet prevalent, broadly-neutralizing antibodies are much wanted but rare. However, little is known about under what conditions such generalists with a high capacity to adapt can be efficiently discovered by evolution. In addition, can epistasis—the source of landscape ruggedness and path constraints—play a different role, if the environment varies in a non-random way? We present a generative model to estimate the propensity of evolving generalists in rugged landscapes that are tunably related and alternating relatively slowly. We find that environmental cycling can substantially facilitate the search for fit generalists by dynamically enlarging their effective basins of attraction. Importantly, these high performers are most likely to emerge at intermediate levels of ruggedness and environmental relatedness. Our approach allows one to estimate correlations across environments from the topography of experimental fitness landscapes. Our work provides a conceptual framework to study evolution in time-correlated complex environments, and offers statistical understanding that suggests general strategies for eliciting broadly neutralizing antibodies or preventing microbes from evolving multi-drug resistance.

## Author summary

Generalists are robust performers under varied environmental conditions, even though they are less fit than specialists in any particular environment. For better (e.g. induction of broadly neutralizing antibody response) or worse (e.g. emergence of multi-drug resistance in microbes), it is important to be able to evolve generalists efficiently. Yet, whether and when environmental changes select generalists over specialists remain largely unknown. Here we develop a dynamic landscape model to study the evolutionary discovery of generalists in time-varying correlated environments. We demonstrate that cycling rugged fitness landscapes can enhance the propensity of evolving fit generalists, via dynamic augmentation of their attractors. We find that high performers are most reliably evolved under intermediate environmental correlations, reflecting a tension between diversity and accessibility. Our approach offers design principles for choosing correlated environments in dynamic protocols to speed or slow generalist evolution in diverse contexts.

## Introduction

Temporally varying environments profoundly influence various properties of evolving systems, including their structure [1–4], robustness [5–8], evolvability [4, 9–11], as well as evolutionary speed [12] and reversibility [13]. Biological populations respond to environmental variations to minimize potential adverse effect on their survival and reproductive growth. Adaptive solutions employed fall into two broad categories: generalists that perform reasonably well across environments, and a diverse mixture of specialists each excelling in a particular environment. Which solution confers the greatest selective advantage in the long run depends on the nature and statistics of environmental variations [14, 15].

Theoretical studies have examined the adaptive utility of survival strategies at different timescales of environmental fluctuations [16–21]. While stochastic switching between distinct specialist phenotypes appears to be favored when environments change sufficiently slowly [16], adopting a single generalist phenotype is shown to be advantageous for rapid fluctuations (e.g. faster than cell division) [22, 23]. Notably, these studies often assume the environments to be unrelated, randomly fluctuating and having few phenotypic dimensions. However, natural environments are often partially related over the course of the system’s adaptation. Furthermore, the high-dimensional evolutionary landscapes, a nonlinear mapping from genotype to function, ultimately guide the adaptive search in the sequence space. Deep mutational scans [24] have mapped out modest-size functional landscapes in fine details for a variety of evolving systems including protein binding affinity [25, 26], enzymatic activity of RNA [27, 28], as well as viral growth [29] and infectivity [30], highlighting the significant role of epistasis—interaction between mutations—in sculpting landscape ruggedness and shaping viable paths of adaptation. Yet, how these intra-landscape structures interplay with inter-landscape correlations to constrain or open pathways toward generalists is not understood.

Generalists can reuse partial solutions in new contexts and hence rapidly adapt to previously unseen environmental conditions [31–33]. In other words, such evolvable solutions are capable of extracting common features from correlated environments. From a landscape perspective, generalist *genotypes* can be recognized as local fitness optima shared by distinct landscapes representing varied environments. Remarkable examples of generalists in adaptive evolution complement each other in an inspiring way: It is desirable for the immune system to evolve broadly neutralizing antibodies [34] that target relatively conserved features of fast evolving pathogens such as HIV and influenza, which can evade recognition by specific antibodies while remaining fit; an unwanted circumstance is that of the emergence of multi-drug resistant bacteria [35] and viruses [36]. Attempts to elicit broad antibody responses and to prevent multi-drug resistance have thus far met with mixed success [37–41], which calls for a better and unified understanding of how evolution discovers generalists in correlated and changing fitness landscapes (or seascapes [18]).

**Fig 1:**
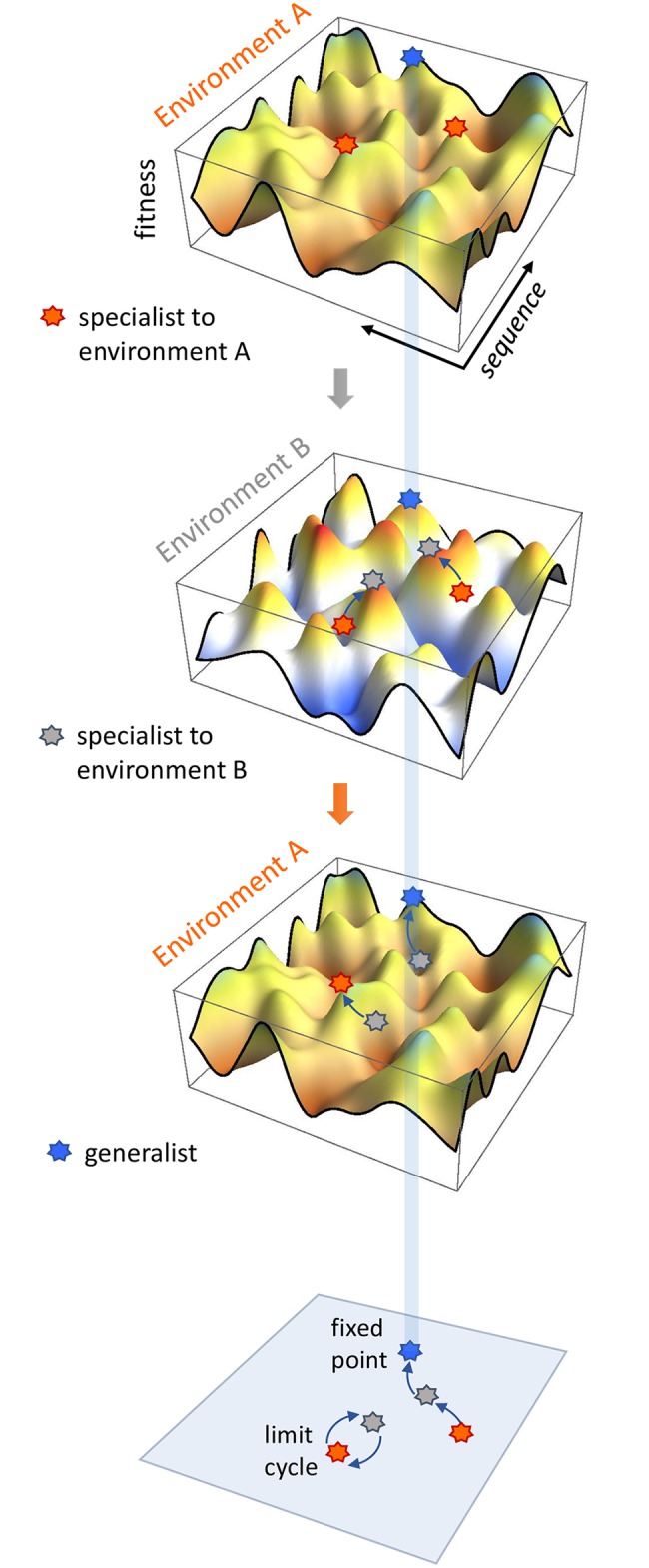
A schematic view of switching landscapes and adaptive walks demonstrating possible attractor types. Cycling distinct yet correlated rugged landscapes can drive population escape from specialists—genotypes fit in a particular environment (orange/gray star in environment A/B) and open new paths (arrows) to generalists—genotypes that remain fit in different environments (blue star). While generalists constitute fixed point attractors in changing environments, specialists located in each other’s basin of attraction on alternating landscapes form limit cycle attractors.

Here we present a general theoretical framework to address the propensity of evolving generalists in high-dimensional environments that are *tunably related* and cycling relatively slowly (Fig 1). This is motivated by evolution of the adaptive immune system against natural pathogens or man-made antigenic stimuli (e.g. vaccines) that change slowly or are sampled sparsely over time, so that considerable immune adaptation occurs in each epoch. Of particular interest are two related questions: (1) Whether and under what conditions can alternating environments grant long-term selective advantage to generalists? (2) How do epistasis and environmental similarity together impact the diversity and accessibility of generalist genotypes?

By constructing and characterizing rugged landscapes with tunable correlations within and between them—a distinctive feature of this work—we find that environmental cycling can substantially enhance the likelihood of evolving fit generalists compared with evolution in a constant environment. Large enhancement requires sufficient, yet not overly strong, similarity in landscape topography, so that cycling can create a pronounced ratchet effect that drives specialists toward generalists. In fact, intermediate environmental relatedness balances a tradeoff between the prevalence and accessibility of generalists; as a result, cycling can preferentially enlarge the attractor region of fit ones. We show in a phase diagram that such balance shifts with the amount of epistasis. This suggests that we may exploit the fitness correlations within and across alternating landscapes to favor the emergence and expansion of fit generalists in a population, against the natural tendency toward evolving specialists in slowly varying environments.

## Results

### Construction of tunably related rugged landscapes

The landscape framework has been used to study physical properties of disordered systems (e.g. macromolecules [42], glasses and spin glasses [43, 44]) as well as nonphysical phenomena ranging from biological evolution [45] to neural computation [46] and business management [47]. The unifying attribute of this framework is its statistical characterization of the global topography of a complex mapping. Interest in fitness landscapes stems from the need for intuition into the evolutionary behavior of populations in the presence of epistasis [48–56]. Epistatic interactions can result in mutations that are individually deleterious but jointly beneficial, giving rise to multiple local optima in a genotypic fitness landscape that represent degenerate solutions to a particular task. Epistasis is central to understanding the predictability of evolutionary paths [57, 58] as well as evolvability [59–61] and adaptation rate [57, 62] of biomolecules.

To determine general properties that arise solely from the global topography of landscapes (i.e., the degree and statistical structure of ruggedness), irrespective of the specific structure of the evolving system *per se*, we use the NK model [63] to represent generic rugged landscapes. This broad family of model landscapes for studying protein evolution describes statistically how adapted states (fitness optima) are organized in the sequence space. NK models have been used to understand antibody affinity maturation, rapid evolution toward higher binding affinity, in a static environment [64–67].

In an NK landscape, the fitness $F(\vec{S})$ of a genotype represented by a bit string $\vec{S}$ of length *N* is defined as the average over the fitness contribution of each bit:

$$\begin{array}{c} F^{\epsilon }\left(\vec{S}\right)=\frac{1}{N}\sum_{i=1}^{N}f_{i}^{\epsilon }\left(S_{i},S_{i_{1}},\cdots ,S_{i_{K}}\right). \end{array}$$ (1)

Here the fitness contribution of bit *i*, $f_{i}^{\epsilon }$, in a given environment *ϵ* depends on $\left\{S_{i},S_{i_{1}},\cdots ,S_{i_{K}}\right\}\equiv {\left\{S\right\}}_{i}$, the state of the *K* + 1 coupled sites influencing the fitness contribution of site *i*. An additive landscape (*K* = 0) has a single global optimum reachable from an arbitrary starting genotype, whereas in a completely random landscape (*K* = *N* − 1) statistical independence of nearby states leads to an extraordinarily rough surface in which on average 2^*N*^/(*N* + 1) local maxima can be surrounded by fitness valleys. Natural populations are likely to be guided by fitness landscapes in between these extremes.

In the case of antibody-antigen binding affinity, each distinct antigen defines a unique hypersurface spanning over a hypercube of 2^*N*^ binary antibody genotypes. To link the level of fitness conservation (the likelihood that a fitness contribution is preserved across environments) to topographical relatedness between landscapes, we consider two environments *A* and *B* in which

$$\begin{array}{c} f_{i}^{B}\left({\left\{S\right\}}_{i}\right)=a_{i}f_{i}^{A}\left({\left\{S\right\}}_{i}\right). \end{array}$$ (2)

This constructs landscape *B* from landscape *A*; the latter is generated according to Eq (1) with $f_{i}^{A}∼U\left(-0.5,0.5\right)$ and randomly chosen interacting neighbors. Statistical properties of landscape topography are insensitive to the distribution of single-site fitness. Although more structured interaction schemes (e.g. block neighborhood) tend to modestly increase ruggedness [56], this factor has little effect on our qualitative results. The strength *a*_*i*_ of correlation between fitness contributions of site *i* in two environments is given by

$$\begin{array}{c} a_{i}=\{\begin{array}{cc} 1 & i\leq n_{p}\\ -1 & i>n_{p} \end{array} \end{array}$$ (3)

In this model, the fitness effect of single mutations under different environments is either conserved (*a*_*i*_ = 1) or subject to tradeoff (*a*_*i*_ = −1). While *n*_*p*_ = *N* corresponds to identical landscapes, *n*_*p*_ = 0 characterizes completely inverted pairs. Therefore, the fraction of conserved fitness contributions, *n*_*p*_/*N*, naturally measures the level of conservation. Furthermore, by making *a*_*i*_ independent of the state of the *K* + 1 epistatically interacting sites, we assume that fitness correlations are preserved in all backgrounds, which decouples the effect of inter-landscape correlations (fitness conservation characterized by *n*_*p*_) from that of intra-landscape correlations (epistasis measured by *K*). This decoupling in turn implies that *n*_*p*_ tunes the topographical similarity without affecting the degree of ruggedness. As a consequence, landscapes thus constructed are tunably related yet statistically equivalent (S1 Fig); changing *n*_*p*_ does not alter the expected number (panel A) and mean fitness (panel B) of local optima, while shifting their locations and modifying topography in their neighborhood.

### Adaptive walks in cycling landscapes

To focus on the effect of global landscape topography on evolutionary dynamics, we consider adaptive walks under strong selection and weak mutation. In this limit, an evolving population can be regarded as a point in the genotype space that moves along paths of increasing fitness in single mutational steps. The population size of interest is sufficiently large to suppress random genetic drift. We further assume “greedy hill climbing” by which any starting genotype can be uniquely associated with a particular fitness peak at the end of the walk. This algorithm thus divides the sequence space into gaplessly packed regions each surrounding a local fitness optimum; these basins of attraction characterize the numeracy of initial states capable of accessing a particular peak via uphill moves.

Natural environments are often partially related over the course of systems adaptation; the very existence of generalists demands a minimal commonality. An immediate consequence of environmental correlation is that different parts of the sequence space may experience different levels of fitness variations as environments cycle: genotypes undergoing large fitness swings correspond to specialists that are fit in a particular environment, whereas genotypes facing small fitness fluctuations represent generalists. Thus, in a landscape description (schematic in Fig 1), generalists can be identified as fitness peaks common to multiple distinct landscapes, whereas specialists correspond to local optima present in a single landscape. Environmental switching opens new possibilities not available in individual landscapes: It can free a population from a specialist peak (red/grey star in environment A/B) and maintain an uphill slope on alternate landscapes, thereby driving population flux (arrows) toward a generalist (blue star in both environments) otherwise inaccessible from a specialist ancestor. Generalists thus act as stationary attractors in changing environments, i.e, fixed points of evolutionary dynamics. Alternatively, specialists located in each other’s basin on alternating landscapes form a limit cycle attractor; as environments alternate, the population passes by each peak cyclically. Therefore, switching environments present two possible classes of attractors (Fig 1 bottom panel): limit cycles among specialists and fixed points at generalists. Tunable correlations (*n*_*p*_ and *K*) control the relative dominance of two attractor types and govern their distribution in sequence space.

We consider the regime in which environmental alternation is sufficiently slow but not too slow, so that in between switches the population is able to reach a local optimum and yet unlikely to escape from it by crossing fitness valleys [68, 69] (see Discussion for further comment on relevant environmental timescales). In this regime, instead of performing an exhaustive study of adaptive dynamics, we directly characterize constituent landscapes and quantify their relationship.

Since our generative model links fitness conservation to topographical similarity, our task now boils down to identifying topographical features that characterize the extent of relatedness, so that these static characteristics can inform the prospects for evolving generalists under alternating environments, including their prevalence, fitness and accessibility. For concreteness, we set *N* = 12 and vary *K* and *n*_*p*_. In all plots, data are averaged over 1000 pairs of landscapes. Error bars are not shown as they are typically very small and have no effect on our results and conclusions.

### Diversity of generalists: Optimum sharing

**Fig 2:**
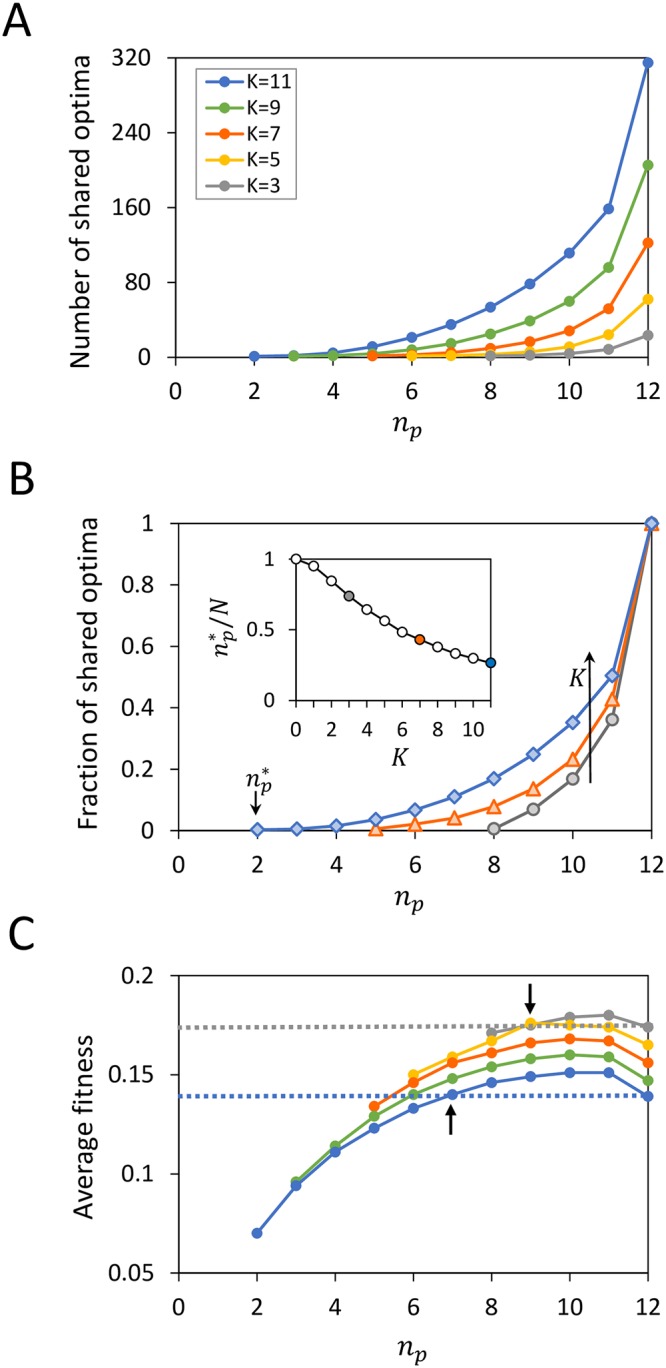
The onset, abundance and fitness of generalists. Expected number (A) and average fitness (C) of shared peaks are shown as a function of the number *n*_*p*_ of conserved fitness contributions at color-coded values of *K*. In (C), dashed lines indicate average fitness of all local optima at *K* = 3 (grey) and *K* = 11 (blue), respectively, which correspond to values at *n*_*p*_ = *N* = 12, whereby all peaks are shared. Above $n_{p}^{\text{min}}$ (marked by arrows) generalists are on average fitter than specialists; this requires a higher level of fitness conservation as ruggedness decreases (e.g. $n_{p}^{\text{min}}=7$ for *K* = 11 whereas $n_{p}^{\text{min}}=9$ for *K* = 3). (B) The fraction of generalists among all fitness peaks increases with *n*_*p*_; $n_{p}^{*}$ indicates the minimum conservation level for generalists to exist. Curves correspond to *K* = 3, 7, 11, increasing in the direction of the arrow. Inset: $n_{p}^{*}/N$ deceases with increasing *K*. Each data point is an average over 1000 landscape pairs.

Local fitness optima that remain in the same location as the environment changes—shared optima across landscapes—represent generalists. Intuitively, as the number *n*_*p*_ of conserved fitness contributions increases, it is more likely that the immediate neighborhood of local peaks remains and hence a greater prevalence of generalists is expected (Fig 2A). Note that the expected number of shared optima between landscapes with equal amounts of conserved and sign-flipped fitness contributions (*n*_*p*_ = *N*/2) is close to that between independent landscapes, so that *n*_*p*_ > *N*/2 (*n*_*p*_ < *N*/2) corresponds to overall positively (negatively) correlated landscapes.

To exclude the effect due to the rapidly growing number of local fitness optima with the size *K* of the epistatic groups, we plot the *fraction* of local optima being shared between landscapes (Fig 2B) and observe two features as *n*_*p*_ increases. First, there is a minimum level of fitness conservation, $n_{p}^{*}/N$, below which no single generalist even exists; in this no-sharing regime, none of the genotypes remains locally optimal as the environment alters, i.e., all adapted states are specialists. Second, both the onset of optimum sharing (at $n_{p}^{*}/N$) and the rate of growth in sharing depend on *K*. In particular, increasing *K* weakens the dependence on *n*_*p*_ of the degree of optimum sharing; as *n*_*p*_ decreases, the fraction of shared optima decreases more slowly at larger *K*. The decline is nearly exponential at *K* ≃ *N*/2 and is faster (slower) than exponential for *K* ≤ (≥)*N*/2. Therefore, stronger ruggedness appears to promote optimum sharing, both by boosting the prevalence of generalists at a given conservation level (Fig 2B, *K* increasing in the direction of the arrow) and by extending their presence to a lower level of fitness conservation (Fig 2B inset).

This finding is somewhat surprising given that increasing ruggedness is often thought to imply reduced fitness correlations, until we realize that the impact of *K* on landscape topography is not merely controlling the abundance of local optima, but also affecting how they are organized in the sequence space. When *K* is small, the highest peaks tend to locate close to one another and the general configuration of the landscape is very non-random. As *K* increases, fit local optima become more evenly distributed which may therefore raise the chance of peak sharing between landscapes. An interesting implication thus follows: while increasing epistasis would reduce fitness correlations within a landscape, it might enhance correlations between landscapes at a given conservation level of fitness contributions.

When are generalists favored over specialists? As known from ecology, generalist birds with intermediate bill lengths may evolve when prey types are alike, whereas specialization develops when more diverse prey types require highly dissimilar beaks [70]. This also applies to the competitive advantage of generalist antibodies over specific ones in recognizing structurally related antigens. Our tunably related NK landscapes capture this trend (Fig 2C): The average fitness of shared optima increases with *n*_*p*_ sublinearly; while in dissimilar environments (small *n*_*p*_) specialists are on average more fit, at sufficiently high levels of environmental relatedness (large *n*_*p*_), generalists become selectively favorable (arrows indicating the crossing between the average fitness of generalists alone, shown in solid lines, and that of specialists and generalists combined, shown in dashed lines). Note that stronger ruggedness enlarges the generalist-favored regime toward a lower conservation level, at the expense of a modest reduction in average fitness.

### Accessibility of generalists: Dynamic basin linking

**Fig 3:**
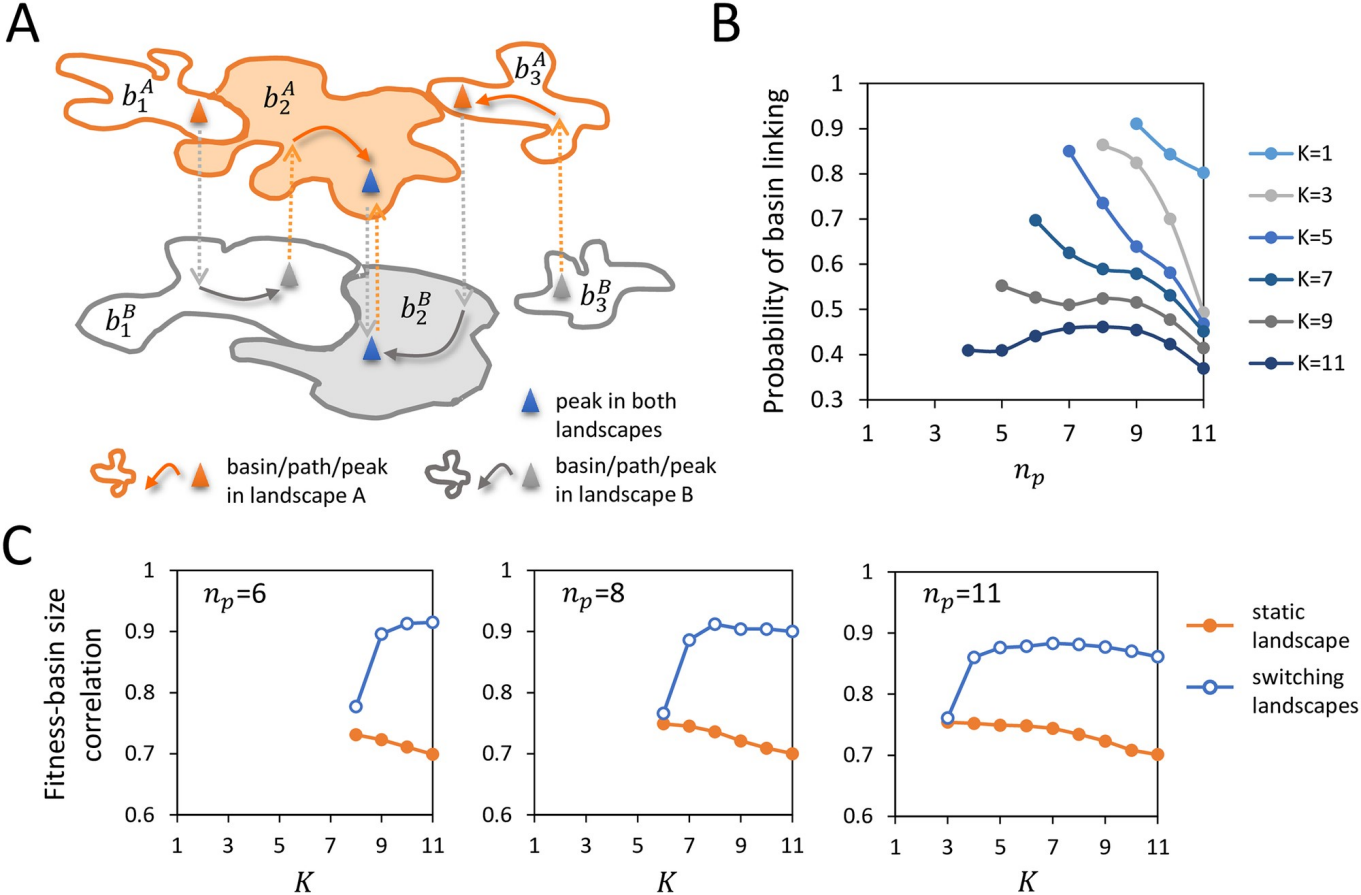
Dynamic basin enlargement via ratchet effects under environmental cycling. (A) Schematically, in a static landscape, only a single basin of attraction (shaded orange/gray in landscape A/B) leads to a generalist (blue peak). Under environmental switches (ABAB or BABA, indicated with dotted arrows), a population initialized in any genotype inside the linked basins (six amorphous shapes with orange or gray borders) can reach the generalist. This chain of basins $\left\{b_{l}^{∊}\right\}$ contains $b_{2}^{A}$ and $b_{2}^{B}$ hinged at the generalist peak, $b_{1}^{A}$ and $b_{3}^{B}$ as two ends, along with $b_{1}^{B}$ and $b_{3}^{A}$ as intermediate links. Thus, the total coverage of linked basins defines the effective accessibility of a generalist in switching landscapes. (B) The fraction of generalists that gain basin size under landscape switching, where *K* increases from top to bottom. Each data point is an average over 1000 realizations of landscape pairs. (C) Correlation between fitness and basin size of local optima in a static landscape (orange filled symbols) and in switching landscapes (blue open symbols).

In an epistatic landscape, provided that fitness conservation is sufficient to support optimum sharing across environments, generalists are likely to be separated from specialists by fitness valleys. If the environment were static, only populations initialized in a single basin of attraction (e.g., $b_{2}^{A}$ or $b_{2}^{B}$ in Fig 3A, the ramified shape filled with orange or gray color) could reach the encompassed generalist peak (blue triangle). In contrast, environmental alternation (dotted arrows) might link to the “hinge” basins ($b_{2}^{A}$ and $b_{2}^{B}$) additional basins that surround specialist peaks (orange or gray triangles) and are otherwise disconnected in individual landscapes. Each successively linked peak is determined as the highest local optimum in current environment that is enclosed by a basin in the preceding environment (e.g., the peak in basin $b_{3}^{A}$ is the fittest genotype in landscape *A* that belongs to basin $b_{2}^{B}$ in landscape *B*). In this way, landscape cycling dynamically enlarges the attractor size of generalists by connecting basins segregated in static environments (e.g. $b_{1}^{B}$ serves as a bridging basin between $b_{1}^{A}$ and $b_{2}^{A}$); such valley-bridging effect of environmental changes has been observed in drug-resistant bacteria [71].

It is important to note that basin linking via landscape switching exemplifies a ratchet effect that enhances population flux from specialists to generalists but not the reverse. In other words, cycling between correlated environments creates an effective continuous positive slope on alternating landscapes toward generalists, because generalists experience smaller variations in their fitness neighborhood than do specialists under environmental switches. Consequently, starting from any genotype inside these dynamically linked basins, a population would converge to the generalist after a sufficient number of switches (*ABAB* or *BABA* in the example in Fig 3A). Therefore, the total coverage of linked basins defines the effective accessibility of a generalist in cycling environments. Note that the number of basins in a chain is relatively modest (e.g. no greater than 8 for *N* = 12; see S2 Fig, panels A and C). Thus, for sequence sizes relevant to antigen or antibiotic binding sites (e.g. of order 10 amino acids), several environmental cycles would suffice to channel the population to generalists.

We next quantify potential benefit of landscape cycling. We first estimate the probability that generalists have greater accessibility under environment cycling than in a static environment. Specifically, we evaluated the fraction of shared optima that have enlarged attractor region via basin linking (Fig 3B). Here results are shown for parameter values where generalists are not too few so that details of landscapes would not cause large variations among realizations. At high levels of fitness conservation (large *n*_*p*_), the frequency of basin linking declines with increasing *n*_*p*_; too similar landscape topography makes it unlikely that a generalist basin in one landscape contains a specialist peak in the other landscape. Increasing epistasis also monotonically diminishes the chance of basin linking; stronger decorrelation in fitness (larger *K*) results in fewer (S2C Fig) and smaller (S2D Fig) linked basins. In highly rugged landscapes (*K* ≥ 9), the likelihood of basin linking exhibits a relatively weak dependence on *n*_*p*_. Intermediate values of *n*_*p*_ result in, on average, longer chains (S2A Fig) of larger basins (S2B Fig) compared to weaker or stronger fitness conservation, reflecting an optimal similarity between landscape profiles that balances the diversity of generalists (degree of optimum sharing) and their accessibility (frequency of basin linking).

To explore how landscape cycling might affect the difficulty in evolutionary search for fit generalists, we computed the correlation coefficient between the fitness and basin size of shared optima (Fig 3C). For a single static landscape, the correlations are already positive and high, decreasing with increasing epistasis, which is consistent with known properties of NK models. Remarkably, under environmental switches, total basin size of linked peaks and their overall fitness are much more correlated compared to the static case; such enhancement in fitness-basin size correlation is significant as long as optimum sharing is prevalent. Strong enhancement occurs at intermediate values of *n*_*p*_, showing little decline toward larger *K*. Taken together, landscape cycling can significantly enlarge the catchment basins of generalists, especially for those with high fitness, well beyond the counterpart in static environments.

### Likelihood of evolving fit generalists

**Fig 4:**
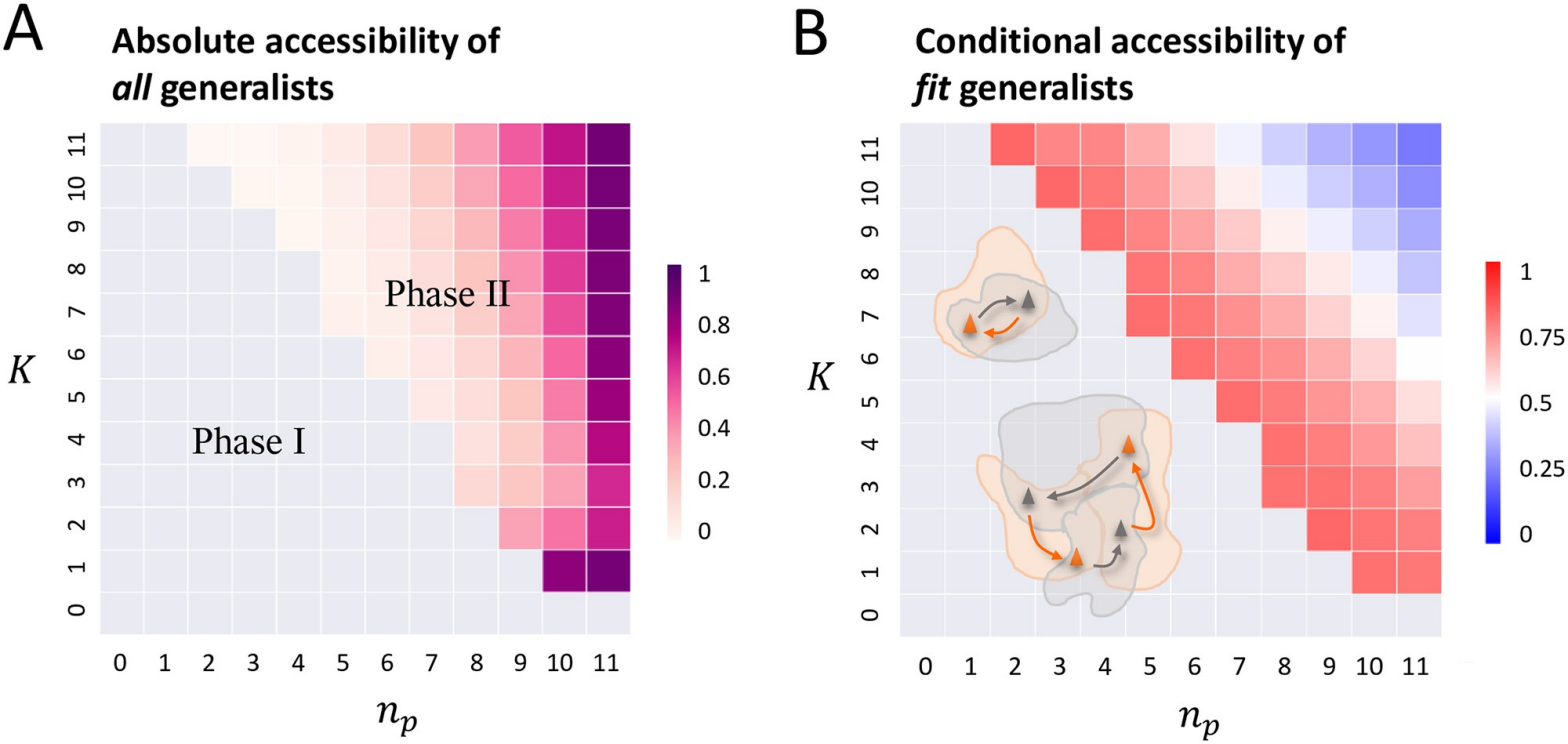
Accessibility of generalists under environmental cycling. (A) The fraction of genotypes that can reach a generalist via an adaptive walk. (B) The ratio of the total basin size of fit generalists (within the top 30% of maximum fitness) to that of all generalists. Both heatmaps are obtained by averaging over 1000 pairs of landscapes at each combination of *n*_*p*_ and *K*. In both diagrams, the gray area corresponds to phase I in which all fitness peaks are specific to one environment; in this no-generalist phase, landscape switching leads to oscillations (panel B, upper inset) or limit cycles (lower inset) among specialists. The colored region represents phase II, where generalists act like hubs into which evolutionary trajectories enclosed by linked basins converge, following multiple landscape switches.

At similar levels of epistasis, landscape topography can nevertheless differ markedly. Whether generalists would benefit from landscape switching depends critically on the topographical relationship between alternating landscapes. As shown in the phase diagrams (Fig 4), for a given *K*, as *n*_*p*_ increases, the system crosses the boundary from phase I in which all adapted states are specialists (grey region) to phase II where generalists constitute stationary attractors in changing environments (colored region). While selectively accessible (i.e. monotonically increasing in fitness) trajectories are rarely circular on a static landscape, closed paths may prevail under environmental cycling in phase I—either due to oscillations between nearby specialists each present in only one landscape (Fig 4B upper inset), or arising from limit cycles composed of specialist peaks located in successive basins on alternating landscapes (Fig 4B lower inset). In both scenarios, the population is driven away from a local optimum upon every switch and never settles. In contrast, in phase II, landscape cycling can channel a population flow to a generalist peak. Note that even in phase II specialists could coexist with generalists, if initially-specialists would generate intermediate products as they transit toward a generalist via linked basins. This is relevant for the makeup of immune repertoires, since instantaneous output (e.g. memory cells and antibodies) accumulate throughout the course of an immune response.

Starting from diverse sequences, how likely will a population discover a generalist under environmental cycling? To estimate the mutational accessibility of generalists (phase II), we first computed the fraction of genotypes that would follow an adaptive path to a shared optimum as landscapes alternate (Fig 4A). This quantity, which measures the overall accessibility of all generalists combined, is large either when the number of shared optima is large at large *n*_*p*_ and large *K* (S3A Fig) or when the basin size of shared optima is large at small *K* (S3C Fig). Yet, among these finally-generalists, the accessibility of the fit ones (defined as being within the top 30% of the maximum fitness among the shared optima) determines the likelihood of evolving fit generalists. Fig 4B shows a heat map of the expected ratio of the total basin size of fit generalist to that of all generalists for each combination of *n*_*p*_ and *K*. This conditional accessibility decreases monotonically as *n*_*p*_ and/or *K* increases, once exceeding the onset of optimum sharing (i.e. in phase II). Notably, the chance of evolving fit generalists is worst in the strong-conservation high-epistasis corner (blue color), where the sequence space divides into many small and rarely linked basins surrounding generalist peaks of which the majority are unfit (S3A and S3B Fig, while the total number of generalists rapidly grows with increasing *n*_*p*_ and *K*, the fit ones saturate in number).

Therefore, to enable efficient discovery of fit generalists, cycling rugged landscapes should have an adequate level of epistasis to allow a diversity of solutions (Fig 2), while presenting complementary profiles of ruggedness to guide adaptation, so that the ratchet effect can most effectively enlarge the set of mutational trajectories leading to fit generalists (Fig 3). Interestingly, the conserved fraction of sitewise fitness contributions—a mean-field-like parameter—closely tunes the topographical correlations between landscapes: Increasing *n*_*p*_ not only increases the number of distinct generalists, but also reduces the average distance between specialists resulting in a lower chance of basin linking. Consequently, an intermediate level of conservation best promotes adaptive linkage of successive specialist basins toward the generalist peak. Moreover, such cycling induced basin linkage preferentially enhances the accessibility of fit generalists (Fig 4B) over less fit ones. Thus, intermediate correlations within and between cycling landscapes strike a balance between diversity and accessibility of fit generalists, facilitating their discovery by evolution.

## Discussion

We present an attempt to endow the ecological notion of generalists with an evolutionary meaning in the context of adaptive strategies in evolving systems. Specifically, we demonstrate the impact that switching between distinct yet related environments might have on the propensity of evolving generalists—genotypes adapted to recurring features in changing environments. We provide a statistical framework to construct and characterize tunably related fitness landscapes, and extend the idea of adaptive walks to study long-term evolution in environments that change on comparable timescales to evolutionary response of a population. We show that landscape topography and relatedness interplay to determine the relative prevalence and fitness of specialist and generalist genotypes. Depending on the degree of fitness conservation and the amount of epistasis, evolutionary dynamics divides into two phases distinguished by the dominant attractor type: (I) oscillations or cycles among specialists in the absence of generalists, and (II) convergence to a generalist after multiple environmental switches. We find that an intermediate amount of epistasis, reflective of evolved functional constraints in biological systems, appears to balance the diversity (Fig 2) and accessibility (Fig 3B) of generalists. What is more, in the convergence phase, an intermediate level of similarity between the structure of ruggedness in alternating landscapes affords the best chance of evolving fit generalists (Fig 4), by more effectively enlarging their basins of attraction (Fig 3B) and strengthening the correlation between basin size and fitness (Fig 3C) compared to weaker or stronger similarity.

In the context of adaptive immunity, recent work has suggested that temporal correlations in antigenic environments crucially regulate evolution of generalist antibodies [72–74]. Here, we make this notion more quantitative: by describing the underlying rugged landscapes in a statistical manner, we turn the abstraction of environmental correlations into concrete measures of relatedness between landscapes, such as the frequency of optimum sharing and dynamic basin linking, and predict adaptive outcomes based on these topographical attributes. Our predictions are relevant because they rely on a statistical framework that neither oversimplifies the topography for interstate dynamics, nor fully characterizes all possible evolutionary trajectories which is not practical for system sizes of most interest. This generic approach thus helps uncover key determinants of the propensity of evolving fit generalists, emphasizing the importance of *simultaneously* considering the role of epistasis and topographical similarity between landscapes in guiding the evolutionary discovery.

The NK model was originally developed for studying antibody affinity maturation in a constant environment. Our extended NK model helps to solve the problems of evolving generalists in time varying correlated environments. By combining laboratory selection experiments with next-generation sequencing, it is now feasible to map sizable fitness landscapes under different environments, e.g. binding of antibodies to different mutants of influenza haemagglutinin [75] and growth of bacteria under different antibiotics [76]. These data could reveal the correlation of empirical fitness landscapes in different environments.

For a combinatorially complete empirical landscape, the fitness contribution of single mutations and the amount of epistasis can be computed by decomposing the landscape using Hadamard-Walsh transform (a generalized class of Fourier transform [52, 77, 78]). *K* can be determined by comparing the contribution of epistasis in the empirical landscape to that in simulated NK landscapes at varied *K* values [79]. Meanwhile, *n*_*p*_ can be estimated by comparing fitness contributions of single mutations across different environments. For example, we performed Hadamard-Walsh transform on fitness landscapes of *β*-lactamase under different *β*-lactam antibiotics [80]. We found that the correlation in fitness effect of single resistance mutations under distinct antibiotics follows a bimodal distribution (i.e. with two peaks around +1 and −1, respectively), suggesting that in this empirical system the effect of single mutations in different environments is predominantly conserved (*a*_*i*_ ∼ 1) or subject to tradeoff (*a*_*i*_ ∼ −1), with more weight on the conserved side (S4 Fig and S1 Text).

If we know more about the mechanism underlying the mutational effects, we can also estimate *n*_*p*_ qualitatively. For example, single amino acid substitutions can affect protein stability and function (e.g. binding affinity to antigens or drugs) [59, 81]. For amino acid sites conserved to ensure structural stability (e.g. in the core of a protein), we expect the fitness effect of mutations to be similar across different environments. In contrast, for sites that determine binding affinity to drugs, we expect the effect of mutations to change drastically for drugs with different binding targets. In the case of multi-drug resistance, we expect drugs with similar resistance profile to have higher *n*_*p*_ than those with dissimilar profile.

Given that we can empirically estimate ruggedness within a landscape and correlations between landscapes, our results (in particular Fig 4B) identify conditions that foster the evolution of fit generalists, as well as strategies to slow their emergence. For the former, in order to efficiently induce generalist antibodies, vaccine antigens that alternate in time should be similar enough to allow generalists to exist and yet sufficiently different so that fit ones own large basins. Furthermore, fitness conservation should be chosen to match the level of epistasis. For instance, antibody binding sites (epitopes) may vary widely in structural complexity and thus differ in *K*. Our finding suggests that evolving antibodies that recognize simple epitopes (small *K*) require switching between similar antigens (large *n*_*p*_), whereas antibodies that bind well to complex epitopes (large *K*) emerge when cycled antigens are sufficiently dissimilar (small *n*_*p*_). Hence, intermediate *n*_*p*_ adjusted to *K* would do best. For the latter, dissimilar antibiotics (small *n*_*p*_ and modest *K*) could avoid generalist microbes and confine the population to a small region of the sequence space under landscape switching (i.e. tight limit cycles), whereas very similar drugs with strong epistasis (large *n*_*p*_ and large *K*) may trap the microbial population to unfit generalist genotypes. In fact, experiment has shown that alternating environments can constrain the evolution of multi-drug resistance, in the regime where *n*_*p*_ is small between different antibiotics [35]. By contrast, for HIV protease inhibitors, *n*_*p*_ is relatively large and hence multi-drug resistance is easily achieved [36]. Thus, our results provide a guide for choosing vaccine antigens (antibiotics) to be cycled in time, so as to promote (suppress) the emergence of fit generalist antibodies (microbes). Similar analysis and principles also apply to cancer treatment [82].

Although the dynamics of adaptation is not explicitly studied here, we speculate that the timescale of environmental changes is important. In particular, the timescale of switching should be intermediate: on the one hand, it must be sufficiently long so a specialist population can reach a nearby fitness peak before the next switch; on the other hand, the epoch should be shorter than the time needed for a generalist population to cross the fitness valley [68, 69]. Therefore, intermediate timescale switching would allow specialists to evolve into generalists but not for generalists to specialize again. Moreover, relevant environmental timescales depend on the degree of correlations, because *n*_*p*_ and *K* can alter the number and distribution of specialists and generalists in sequence space. Hence, different parts of the parameter space may entail different favorable timescales for evolving generalists. For example, a few long cycles would suffice at small *K* and large *n*_*p*_, whereas many relatively brief cycles are needed for large *K* and small *n*_*p*_. In future work, we will study explicitly the impact of switching rates on evolutionary outcomes in finite populations. In contrast to very fast or very slow switching that can be understood as effective static environments, we speculate, as indicated by studies in other contexts [17, 83, 84], that cycling at intermediate timescales can drive evolution toward novel nonequilibrium states [18, 85–87] and are key to evolving generalists.

Another interesting direction is generalization of our findings to evolution under complex schemes of switching environments (e.g. alternating among more than two landscapes). As the variety of distinct environments increases, the possibilities of switching schemes expand quickly, since it permits combinatorial ways of arranging successive environments and wide choices for the timing of switches. We expect, within our model, that generalists will likely become rarer given more diverse environments. And yet, guiding principles derived from this work still hold in choosing *successive pairs* of environments in a temporal sequence so as to enlarge basins of fit generalists; further, the underlying mechanism that evolutionary ratchets are most effective at intermediate correlations remains valid. In this sense, our work complements prior studies on switching schemes, e.g., in reversal of drug resistance [88] and anti-cancer therapies [89], which are based on system-specific exhaustive approaches. Sequential protocols have also been investigated in the context of logic circuits and RNA secondary structure [33]. While a sequence of modularly varying environments is favored over a fixed environment in driving evolution of useful novel phenotypes, tuning the level of correlation within and between environments explored here may further broaden the range of strategies.

Finally, our results based on temporally varying environments can be extended to understand adaptation in spatially heterogeneous environments, where landscape switching arises from migration between distinct yet connected habitats or microenvironments [90, 91].

## Methods

Consider two environments A and B. Each environment defines a fitness landscape in the sequence space, characterized by a set of local optima (fitness peaks), $\left\{{\vec{s}}_{j}^{∊}\right\}$, and associated basins of attraction, $\left\{b_{j}^{∊}\right\}$. Here *ϵ* labels the environment and *j* runs through in total $N_{\text{opt}}^{∊}$ local optima in environment *ϵ*. Generalists correspond to local optima common to different environments and are represented by $\left\{{\vec{s}}_{l}\right\}$, l∈G, where G is a set of size $N_{g}$ containing the sequence labels of generalist genotypes. The rest of the local optima are unique to a particular environment and thus represent specialists. As the amount of epistasis (*K*) and the level of fitness conservation (*n*_*p*_) vary, both the number and the distribution of generalists and specialists in sequence space will change. Below we introduce the measures to quantify the prevalence and accessibility of generalists in static and switching environments.

### Diversity of generalists

We calculate the fraction of fitness optima shared between environments (Fig 2B), denoted by *θ*_g_, as the ratio of the number of generalists ($N_{g}$) to the environment-averaged number of all local optima (${\bar{N}}_{\text{opt}}^{∊}$):

$$\begin{array}{c} \theta_{g}=\left\langle \frac{N_{g}}{{\bar{N}}_{\text{opt}}^{\epsilon }}\right\rangle . \end{array}$$ (4)

Here and below we use the overbar to indicate average over environments and use the angular bracket to represent ensemble average over many landscapes for given *K* and *n*_*p*_.

### Accessibility of generalists

#### Dynamic basin linking

We identify chains of linked basins under landscape switching as follows: Starting in environment A, we search through the basin of a generalist. If it contains any fitness peak in environment B, pick the fittest one and store it as a linked peak and its basin (in environment B) a linked basin. We then search through this linked basin for fitness peaks in environment A; if identified, we link the fittest peak and its basin (in environment A). Iterate until the newly linked basin no longer contains any peak in the other environment. Repeating this process starting from environment B, we obtain a full chain of linked basins hinged at the generalist (illustrated in Fig 3A).

Note that this is an approximation of the full basin enlargement enabled by environmental cycling, because we extend the chains only with the highest peak in each successive environment, ignoring the contribution of less fit peaks. Yet this is a good approximation in the limit of strong selection as assumed in this study. In addition, non-greedy paths neglected here may divert part of the population from the paths toward the generalist. Explicit stochastic simulations of population trajectories would help quantify these effects.

#### Basin size

The basin size *V*^*ϵ*^ $\left({\vec{s}}_{j}^{∊}\right)$ of a fitness peak ${\vec{s}}_{j}^{∊}$ in environment *ϵ* is computed as the number of starting sequences that can reach ${\vec{s}}_{j}^{∊}$ via a greedy walk, which is composed of uphill moves on the landscape with the largest available fitness increase in each step.

The effective basin size $\tilde{V}\left({\vec{s}}_{l}\right)$ of a generalist ${\vec{s}}_{l}$ in alternating environments is defined as the total size of linked basins in the chain, with the overlapping portion of adjacent basins counted once.

#### Probability of basin linking

We measure the probability of basin linking under alternating environments (Fig 3B) as follows:

$$\begin{array}{c} P(\tilde{V}>V)=\left\langle \frac{N_{g}\left(\tilde{V}>V\right)}{N_{g}}\right\rangle . \end{array}$$ (5)

Here the numerator counts the number of generalists that have enlarged basin size in switching landscapes ($\tilde{V}$) than in a static landscape (*V*); these generalists gain linkage to additional specialist basins under cycling.

#### Correlation between fitness and basin size

We quantify how well fitness, *F*, and basin size, *V*, are correlated in static and cycling environments (Fig 3C) using the following metrics:

In a static environment *ϵ*,

$$\begin{array}{c} C(F^{\epsilon },V^{\epsilon })=\left\langle \frac{m\left(F^{\epsilon }V^{\epsilon }\right)-m\left(F^{\epsilon }\right)m\left(V^{\epsilon }\right)}{\sqrt{v\left(F^{\epsilon }\right)v\left(V^{\epsilon }\right)}}\right\rangle \end{array}$$ (6)

where we define $m\left(X^{∊}\right)=\sum_{j=1}^{N_{\text{opt}}^{∊}}X^{∊}\left({\vec{s}}_{j}^{∊}\right)/N_{\text{opt}}^{∊}$ and *v*(*X*^*ϵ*^) = *m*(*X*^*ϵ*^ *X*^*ϵ*^) − *m*(*X*^*ϵ*^)*m*(*X*^*ϵ*^) to compute mean and variance respectively. The correlation strength is then averaged over environments.

In cycling environments,

$$\begin{array}{c} C\left(\tilde{F},\tilde{V}\right)=\left\langle \frac{m\left(\tilde{F}\tilde{V}\right)-m\left(\tilde{F}\right)m\left(\tilde{V}\right)}{\sqrt{v\left(\tilde{F}\right)v\left(\tilde{V}\right)}}\right\rangle \end{array}$$ (7)

where we define functions $m\left(\tilde{X}\right)=\sum_{l=1}^{N_{g}}\tilde{X}\left({\vec{s}}_{l}\right)/N_{g}$ and $v\left(\tilde{X}\right)=m\left(\tilde{X}\tilde{X}\right)-m\left(\tilde{X}\right)m\left(\tilde{X}\right)$. Here $\tilde{F}$ represents the overall fitness of linked peaks under landscape cycling for a given generalist and $\tilde{V}$ denotes its effective basin size, i.e., the total sequence-space “volume” of linked basins.

### Likelihood of evolving fit generalists

The absolute accessibility of all generalists (Fig 4A), denoted by *A*_0_, is defined as the total coverage of generalists’ effective basins in switching landscapes:

$$\begin{array}{c} A_{0}=\left\langle \sum_{l\in G}\tilde{V}\left({\vec{s}}_{l}\right)\right\rangle /2^{N}. \end{array}$$ (8)

The conditional accessibility of fit generalists (Fig 4B), denoted by *A*_*c*_, is measured by the total effective basin size of generalists within the top 30% of maximum fitness, normalized by the total basin size of all generalists combined:

$$\begin{array}{c} A_{c}=\left\langle \sum_{l\in G}\tilde{V}\left({\vec{s}}_{l}|{\bar{F}}^{\epsilon }\left({\vec{s}}_{l}\right)\geq 70\%\times \max_{l}{\bar{F}}^{\epsilon }\left({\vec{s}}_{l}\right)\right)/\sum_{l\in G}\tilde{V}\left({\vec{s}}_{l}\right)\right\rangle \end{array}$$ (9)

where ${\bar{F}}^{∊}\left({\vec{s}}_{l}\right)$ indicates environment-averaged fitness of a generalist ${\vec{s}}_{l}$.

## Supporting information

S1 Text Fourier analysis of fitness landscapes of TEM-*β* lactamase under different antibiotics. (PDF)

S1 Fig Statistical equivalence of tunably related landscapes. (A) Total number of local optima. (B) Average fitness of local optima. Almost complete overlap of data points at different values of *n*_*p*_ (different symbols) indicates that changing *n*_*p*_ does not alter the number and average fitness of local optima for a given number *K* of interacting sites. Each data point is an average over 1000 landscapes.
(PDF)

S2 Fig Characteristics of dynamic basin linking. Histograms of the number (left column) and average size (right column) of linked basins under landscape switching for *K* = 9 (upper row) and *n*_*p*_ = 8 (lower row). The number of linked basins excludes the one associated with the shared optimum. For each combination of *n*_*p*_ and *K*, around 200 generalists are collected. (A and B) With relatively strong epistasis (*K* = 9), an intermediate level of fitness conservation (*n*_*p*_ = 8, orange bars) leads to longer chains of linked basins (A) of larger average size (B), compared to weaker or stronger conservation. Inset of A: the weight of the *n*_*p*_ = 8 term for various numbers of linked basins; the grey line marks 1/3. Inset of B: the average size of linked basins at different values of *n*_*p*_. (C and D) At an intermediate level of fitness conservation (*n*_*p*_ = 8), stronger epistasis (larger *K*) results in shorter chains of linked basins (C) of significantly smaller average size (D).
(PDF)

S3 Fig Diversity and accessibility of generalists. The number (left column) and average basin size (right column) of all generalists (A, C) and fit generalists (B, D, within top 30% of maximum fitness among all local optima). All color-coded values are in logarithmic scales and averaged over 1000 landscape pairs for each combination of *n*_*p*_ and *K*. While the number of generalists rapidly grows with *n*_*p*_ and *K* (A), the number of fit ones saturates (B). Average basin sizes of generalists decrease as *K* increases and are largest at intermediate *n*_*p*_ for large *K*, consistent with the trend of the probability of basin linking (Fig 3B, main text).
(PDF)

S4 Fig Correlation in fitness effect of single resistance mutations under different antibiotics. (A) Fitness effect of single drug-resistance mutations, i.e., first-order Fourier coefficients, is found to be predominantly conserved (Pearson correlation close to 1) or anticorrelated (Pearson correlation close to −1) under pairs of different drugs. The data of empirical fitness landscapes under 15 different *β*-lactam antibiotics are taken from Table in Ref. [80]. (B) Correlation in fitness effect of single mutations between random fitness landscapes does not show a bimodal distribution. 100 random fitness landscapes are generated that have the same size as the empirical fitness landscapes (*L* = 4, 16 genotypes).
(PDF)
