# Supplementary material for: Evolving generalists in switching rugged landscapes
Source: PLoS Comput Biol. 2019 Oct 1;15(10):e1007320. doi: 10.1371/journal.pcbi.1007320 (PMC6771975; doi:10.1371/journal.pcbi.1007320)
Supplement: S1 Text — (PDF) [file pcbi.1007320.s001.pdf]

Fourier analysis of fitness landscapes of TEM-β lactamase under different antibiotics

We decomposed the fitness landscape into epistatic interaction of different orders by a generalized class of Fourier analysis, known as Walsh transform or Hadamard transform. The Fourier coefficients given by the transform can be interpreted as epistasis of different orders.

We applied Fourier analysis to fitness landscapes of TEM-β lactamase under 15 different antibiotics (Ref [80] in the main text). Each fitness landscape consists of 16 genotypes and can be decomposed into Fourier coefficients of 1st order (4 coefficients), 2nd order (6 coefficients), 3rd order (4 coefficients), and 4th order (1 coefficient). The 1st order coefficients represent fitness effects of single mutations, the 2nd order coefficients represent pairwise interactions, etc.

We found that the correlation between 1st order Fourier coefficients of landscapes under different antibiotics follows a bimodal distribution (i.e. with two peaks around +1 and −1, respectively), suggesting that in this empirical system the effect of single mutations in different landscapes is predominantly conserved (+1) or subject to tradeoff (−1) (Supplementary Figure S4A). The bimodality is not observed for simulated random fitness landscapes (Supplementary Figure S4B).

For a binary sequence $\vec{z}$ with dimension L (z_i is 1 if a mutation is present at position i and 0 otherwise), the Fourier decomposition theorem states that the fitness function $f(\vec{z})$ can be expressed as

$$f(\vec{z}) = \sum_{\vec{k}} \hat{f}_{\vec{k}} (-1)^{\vec{z}\cdot\vec{k}}. \tag{1}$$

The formula for the Fourier coefficients $\hat{f}_{\vec{k}}$ is given by

$$\hat{f}_{\vec{k}} = \frac{1}{2^L} \sum_{\vec{z}} f(\vec{z}) (-1)^{\vec{z}\cdot\vec{k}}. \tag{2}$$

For example, we can expand the fitness landscape up to the second order,

$$f(\vec{\sigma}) = \hat{f}_0 + \sum_i \hat{f}_{\vec{e_i}} \sigma_i + \sum_{i<j} \hat{f}_{\vec{e_i}+\vec{e_j}} \sigma_i \sigma_j + \cdots \tag{3}$$

where $\sigma_i \equiv (-1)^{z_i} \in \{+1, -1\}$, $\vec{e_i}$ is a unit vector along the ith direction. In our analysis of subgraphs, there are in total $2^4 = 16$ terms in the Fourier decomposition, with $\binom{4}{n}$ terms at the nth order ($n = 0, 1, 2, 3, 4$).
