# Supplementary figures and images for: Evolving generalists in switching rugged landscapes
Source: PLoS Comput Biol. 2019 Oct 1;15(10):e1007320. doi: 10.1371/journal.pcbi.1007320 (PMC6771975; doi:10.1371/journal.pcbi.1007320)

A

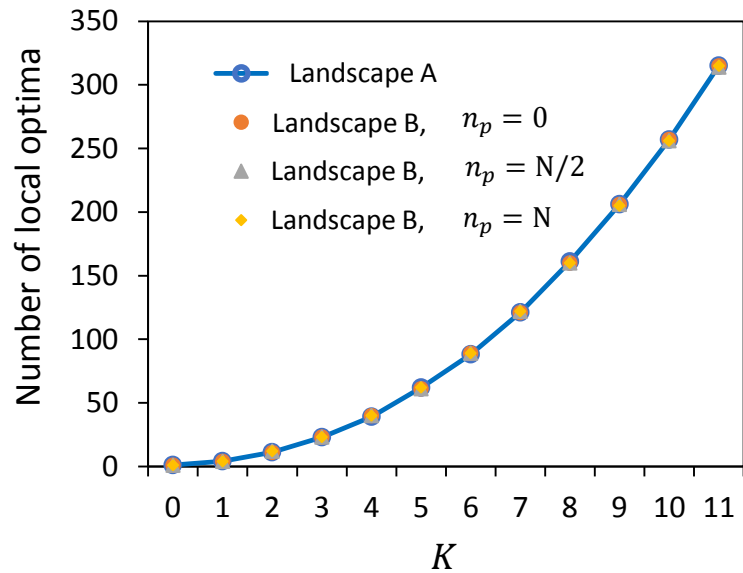

B

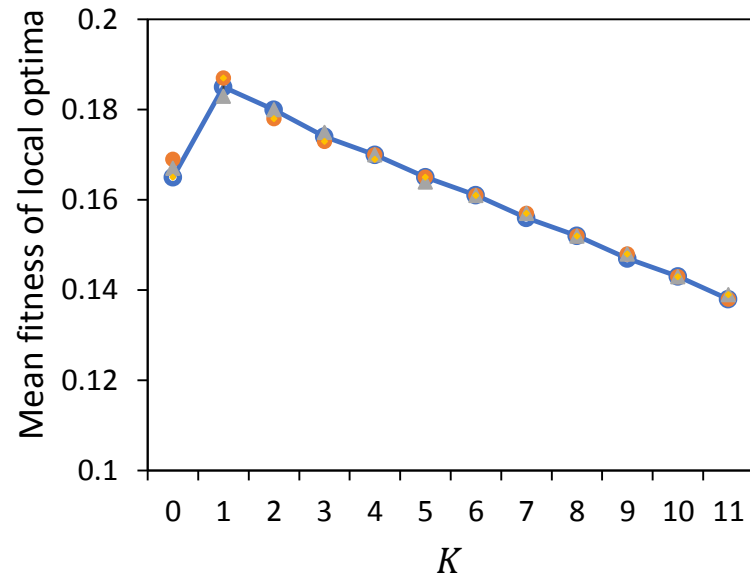

Supplement: S1 Fig — (A) Total number of local optima. (B) Average fitness of local optima. Almost complete overlap of data points at different values of np (different symbols) indicates that changing np does not alter the number and average fitness of local optima for a given number K of interacting sites. Each data point is an average over 1000 landscapes. (PDF) [file pcbi.1007320.s002.pdf]

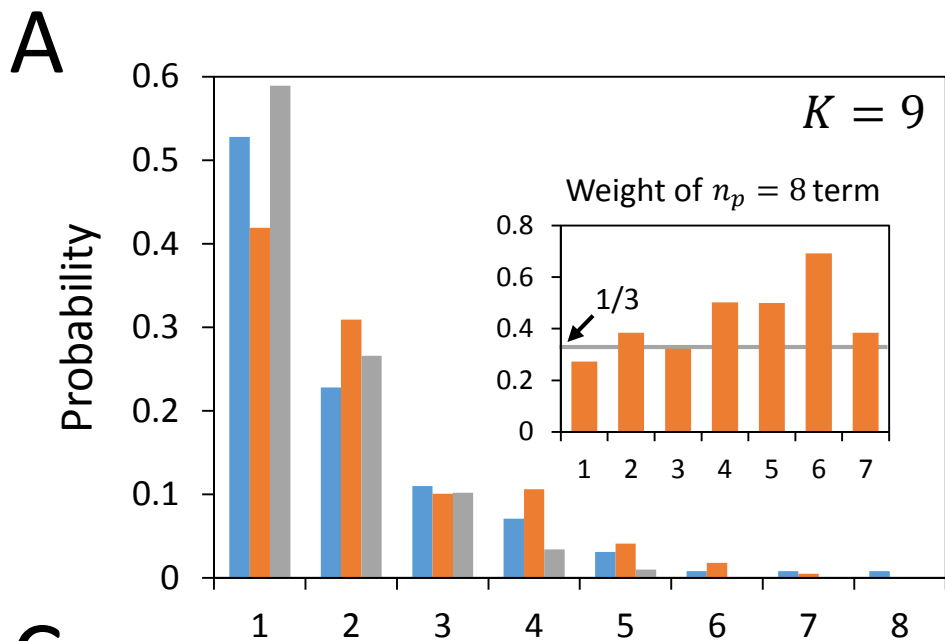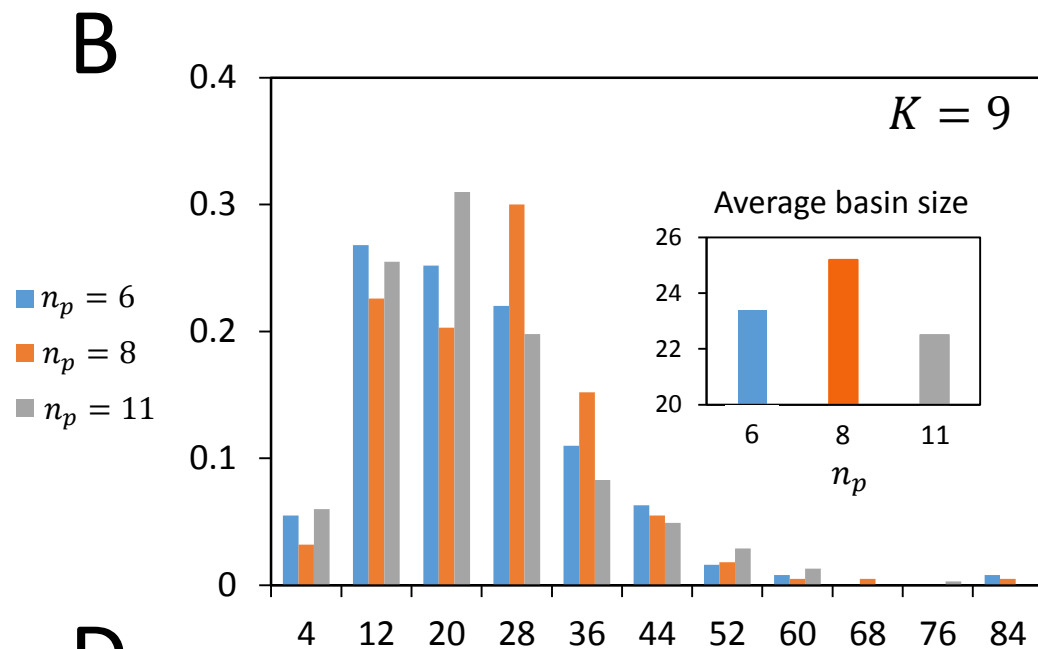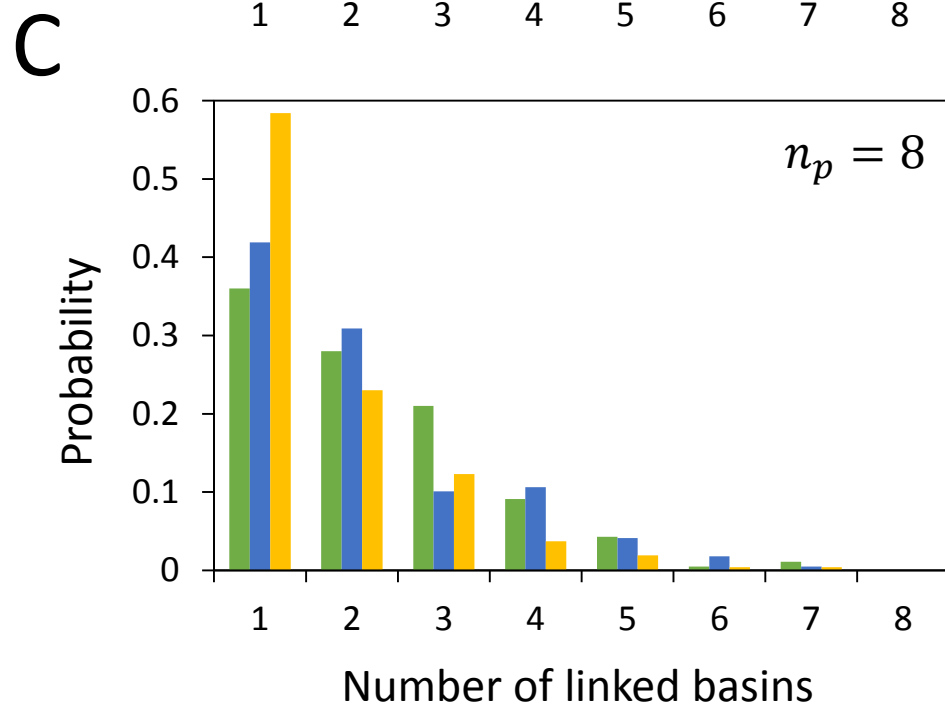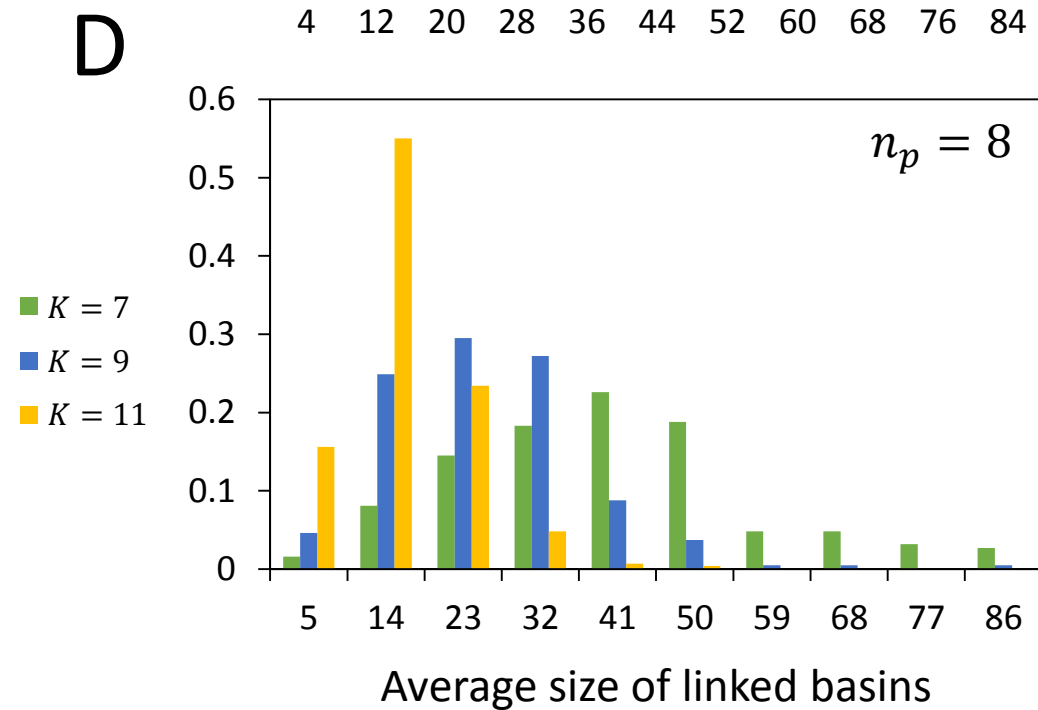

Supplement: S2 Fig — Histograms of the number (left column) and average size (right column) of linked basins under landscape switching for K = 9 (upper row) and np = 8 (lower row). The number of linked basins excludes the one associated with the shared optimum. For each combination of np and K, around 200 generalists are collected. (A and B) With relatively strong epistasis (K = 9), an intermediate level of fitness conservation (np = 8, orange bars) leads to longer chains of linked basins (A) of larger average size (B), compared to weaker or stronger conservation. Inset of A: the weight of the np = 8 term for various numbers of linked basins; the grey line marks 1/3. Inset of B: the average size of linked basins at different values of np. (C and D) At an intermediate level of fitness conservation (np = 8), stronger epistasis (larger K) results in shorter chains of linked basins (C) of significantly smaller average size (D). (PDF) [file pcbi.1007320.s003.pdf]

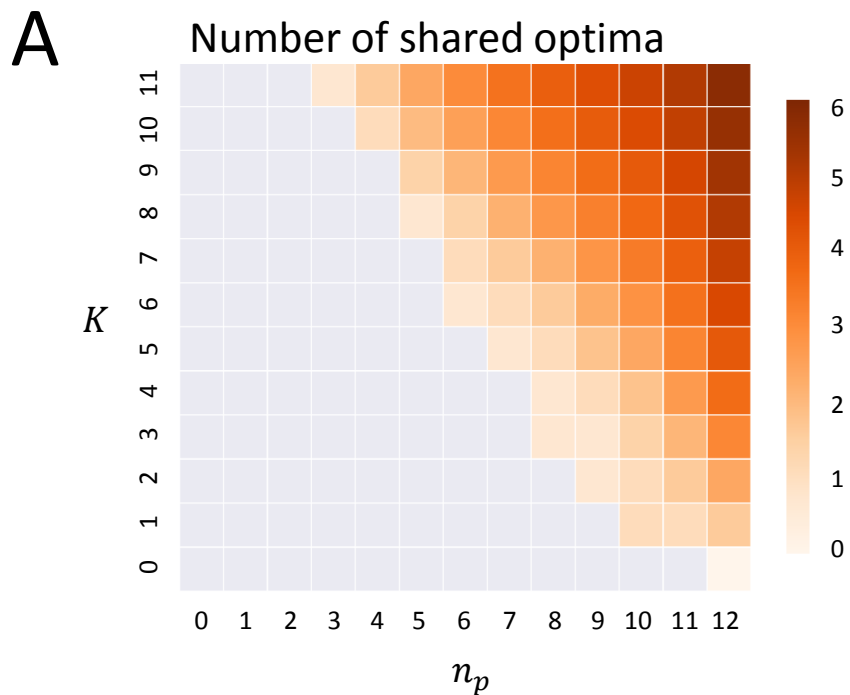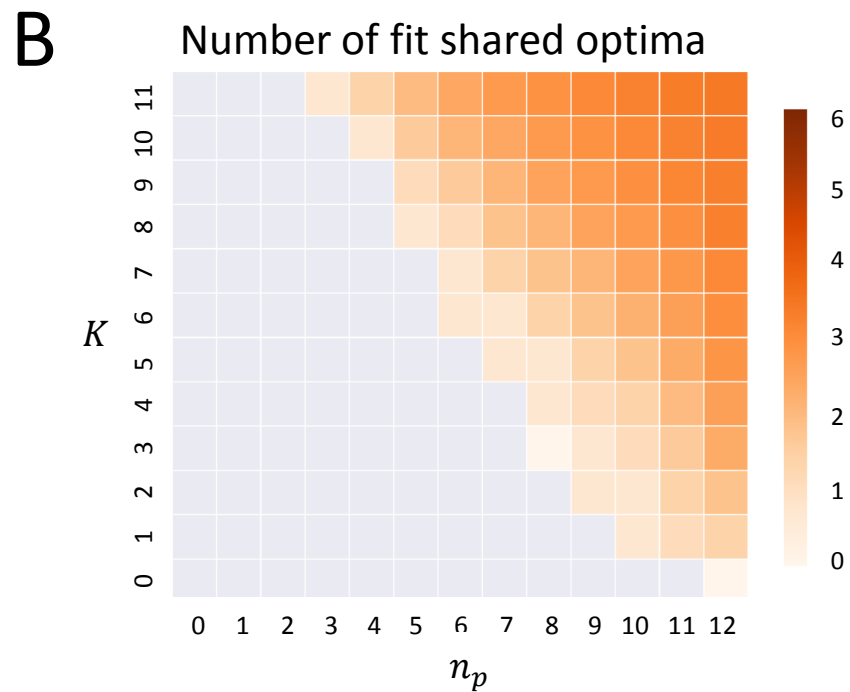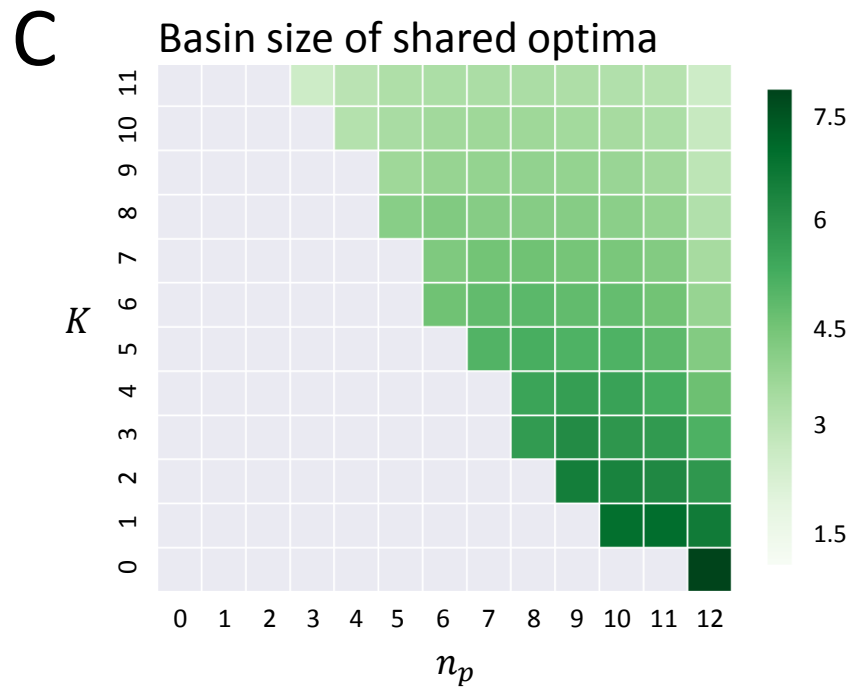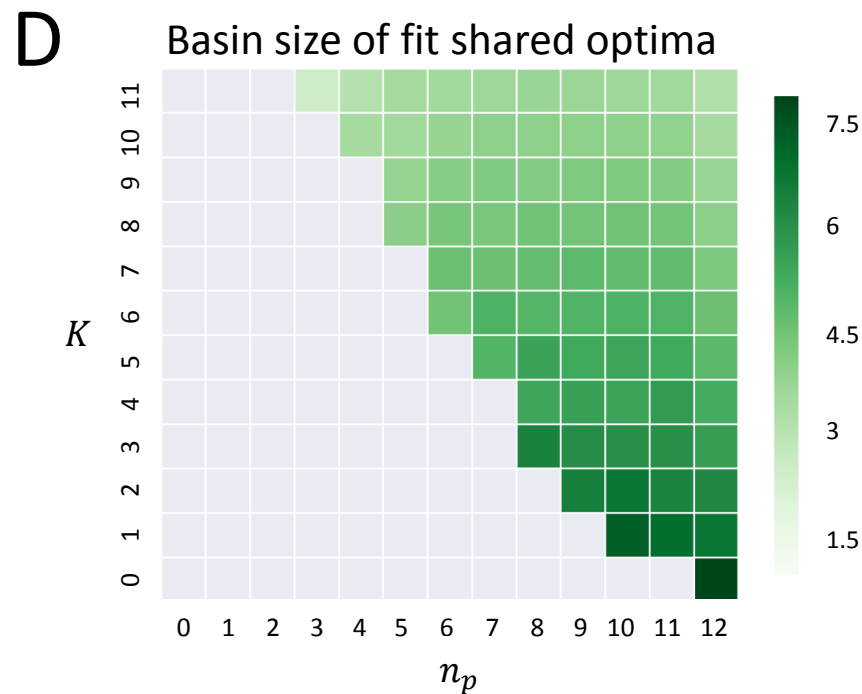

Supplement: S3 Fig — The number (left column) and average basin size (right column) of all generalists (A, C) and fit generalists (B, D, within top 30% of maximum fitness among all local optima). All color-coded values are in logarithmic scales and averaged over 1000 landscape pairs for each combination of np and K. While the number of generalists rapidly grows with np and K (A), the number of fit ones saturates (B). Average basin sizes of generalists decrease as K increases and are largest at intermediate np for large K, consistent with the trend of the probability of basin linking (Fig 3B, main text). (PDF) [file pcbi.1007320.s004.pdf]

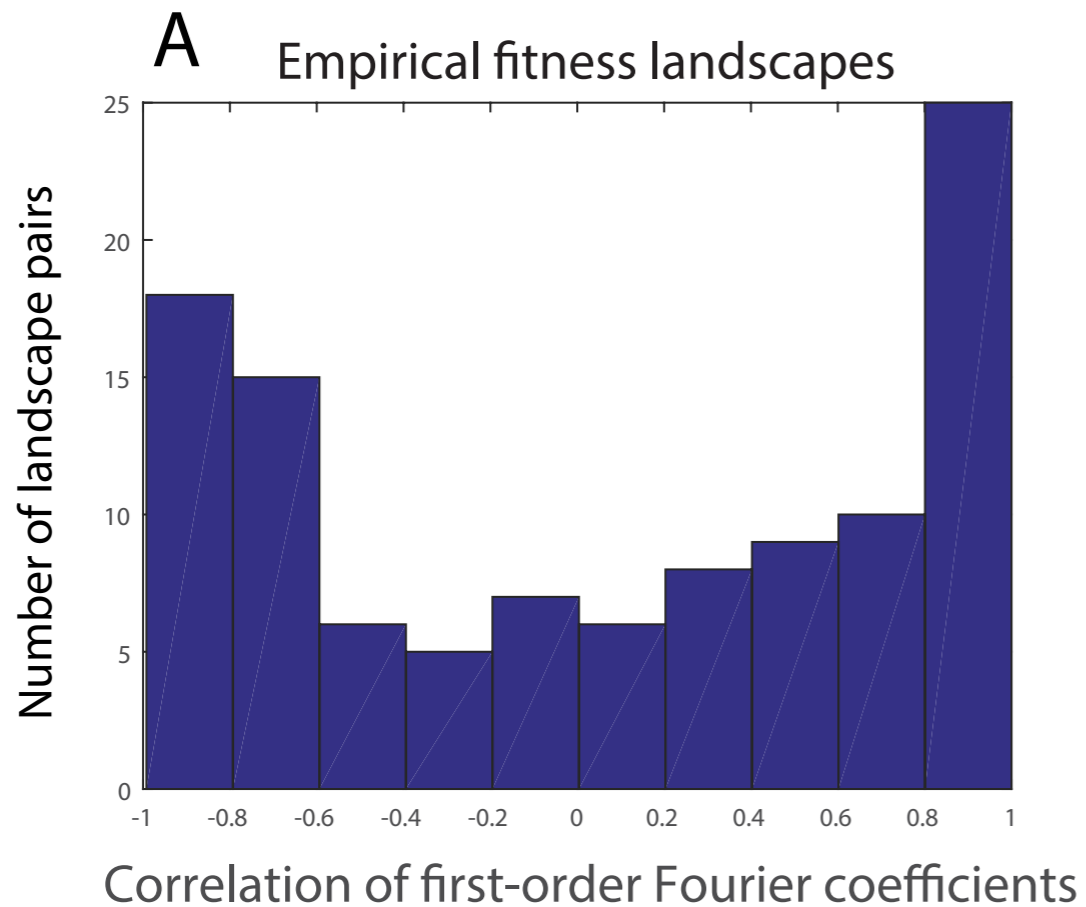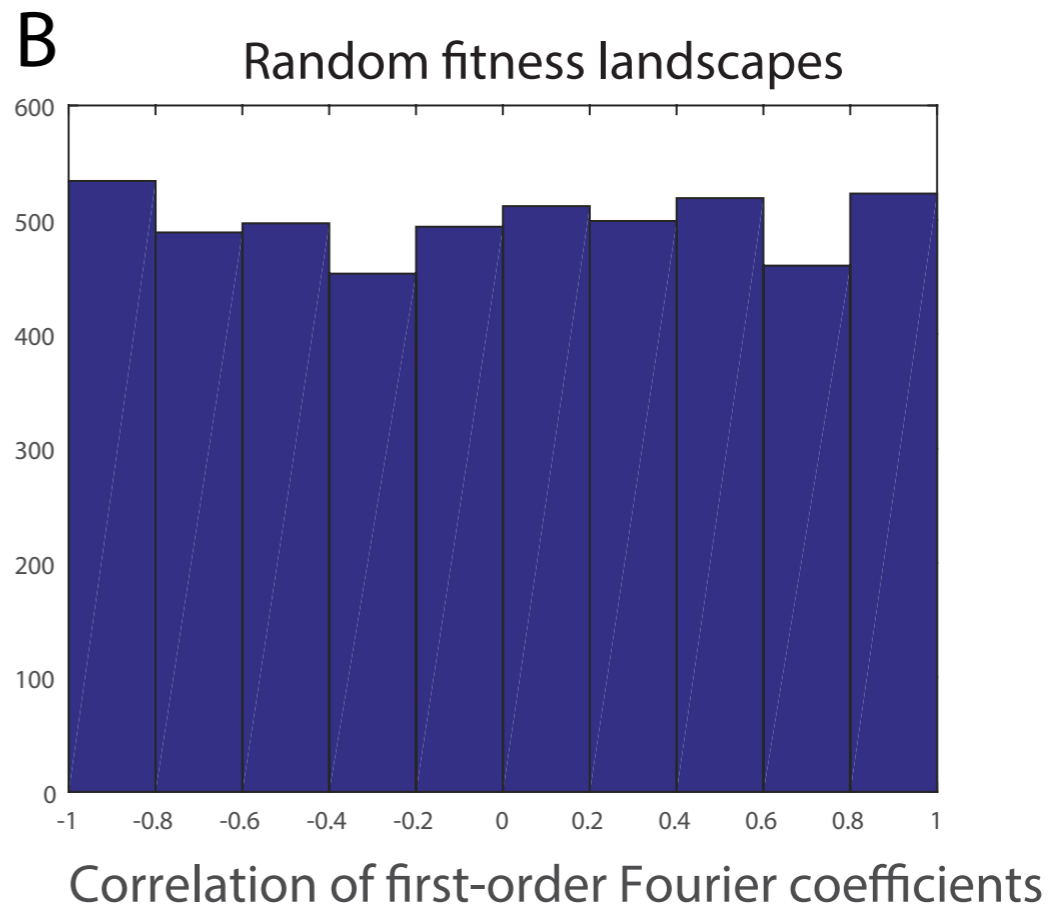

Supplement: S4 Fig — (A) Fitness effect of single drug-resistance mutations, i.e., first-order Fourier coefficients, is found to be predominantly conserved (Pearson correlation close to 1) or anticorrelated (Pearson correlation close to −1) under pairs of different drugs. The data of empirical fitness landscapes under 15 different β-lactam antibiotics are taken from Table in Ref. [80]. (B) Correlation in fitness effect of single mutations between random fitness landscapes does not show a bimodal distribution. 100 random fitness landscapes are generated that have the same size as the empirical fitness landscapes (L = 4, 16 genotypes). (PDF) [file pcbi.1007320.s005.pdf]
